# Differences in Longitudinal Fasting Blood Glucose and Mortality Risk Across the Lifespan Between Mice and Humans

**DOI:** 10.1093/geroni/igaa057.1672

**Published:** 2020-12-16

**Authors:** Dushani Palliyaguru, Eric Shiroma, John Nam, Luigi Ferrucci, Rafael de Cabo, SLAM Investigators

**Affiliations:** National Institute on Aging, Bethesda, Maryland, United States

## Abstract

Aging profoundly affects metabolism where trajectories of metabolic indices serve as strong predictors of health, disease and mortality. Mice and non-human primates are widely used to model all aspects of human biology, including metabolism. However, there is limited knowledge on how different species metabolically age during their life course. Here, we compare longitudinal predictors of health and mortality of three major metabolic indices among mice, non-human primates and humans. Longitudinal fasting blood glucose, body weight and body composition over the lifespan were compared across species in mice (Study of Longitudinal Aging in Mice), Rhesus monkeys (NIA and Wisconsin colonies) and humans (Baltimore Longitudinal Study on Aging). Survival analysis was conducted to calculate the risk of death for subjects with highest and lowest quartiles of fasting blood glucose. We will present data highlighting species-specific mechanisms of glucose homeostasis over the lifespan and its association with mortality.

